# Mucosal leishmaniasis mimicking T-cell lymphoma in a patient receiving monoclonal antibody against TNFα

**DOI:** 10.1371/journal.pntd.0005807

**Published:** 2017-09-21

**Authors:** Antonio Carlos Nicodemo, Daniel Fernandes Duailibi, Diego Feriani, Maria Irma Seixas Duarte, Valdir Sabbaga Amato

**Affiliations:** 1 Department of Infectious Diseases, University of São Paulo Medical School, Sao Paulo, Brazil; 2 Department of Infectious and Parasitic Diseases, Clinical Hospital, University of São Paulo Medical School, Sao Paulo, Brazil; 3 Department of Pathology, University of São Paulo Medical School, Sao Paulo, Brazil; Ben-Gurion University of the Negev, ISRAEL

## Case report

**Fig 1:**
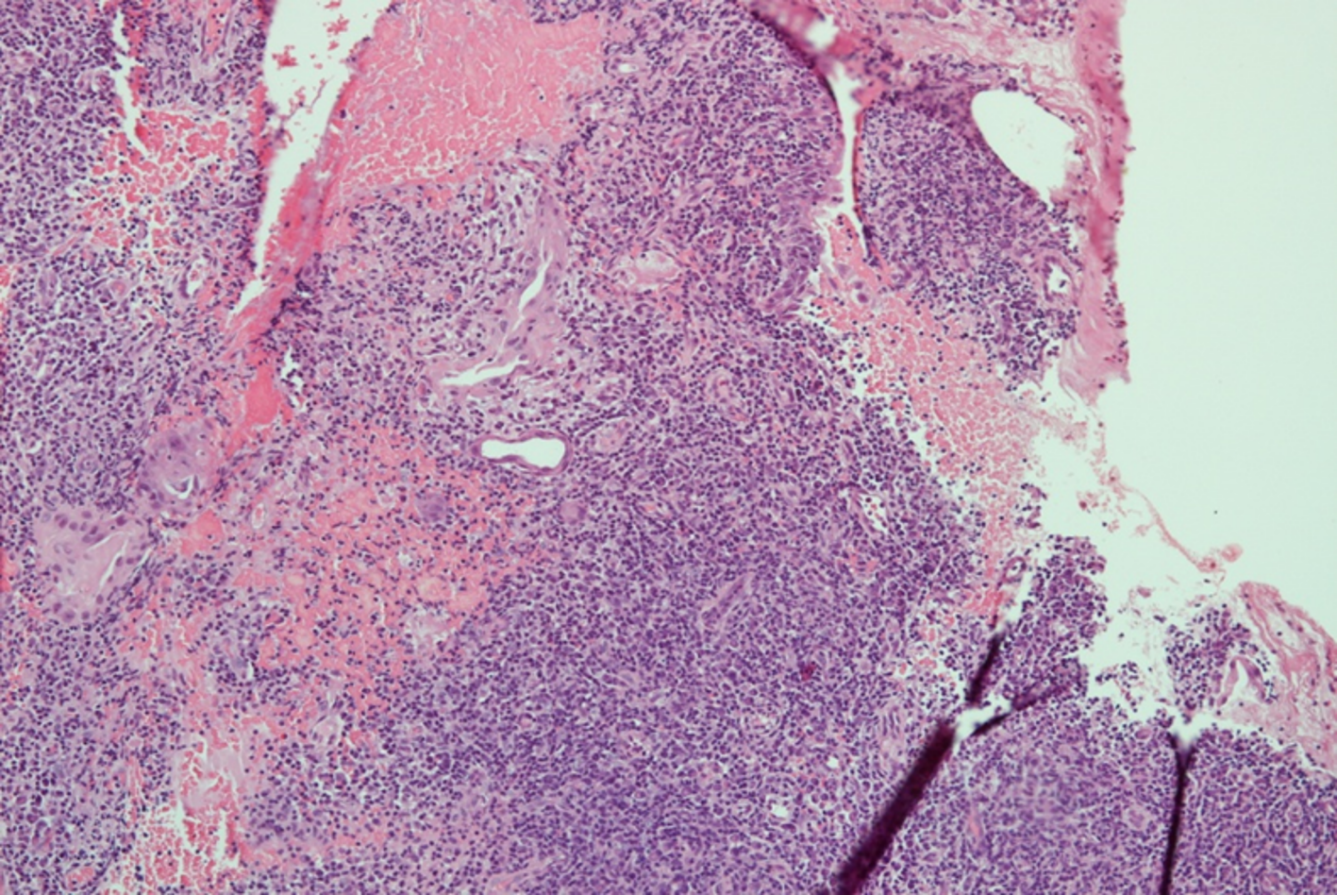
Hematoxylin–eosin revealing lymphoplasmacytic infiltrate with some areas of necrosis and mucosal ulceration.

**Fig 2:**
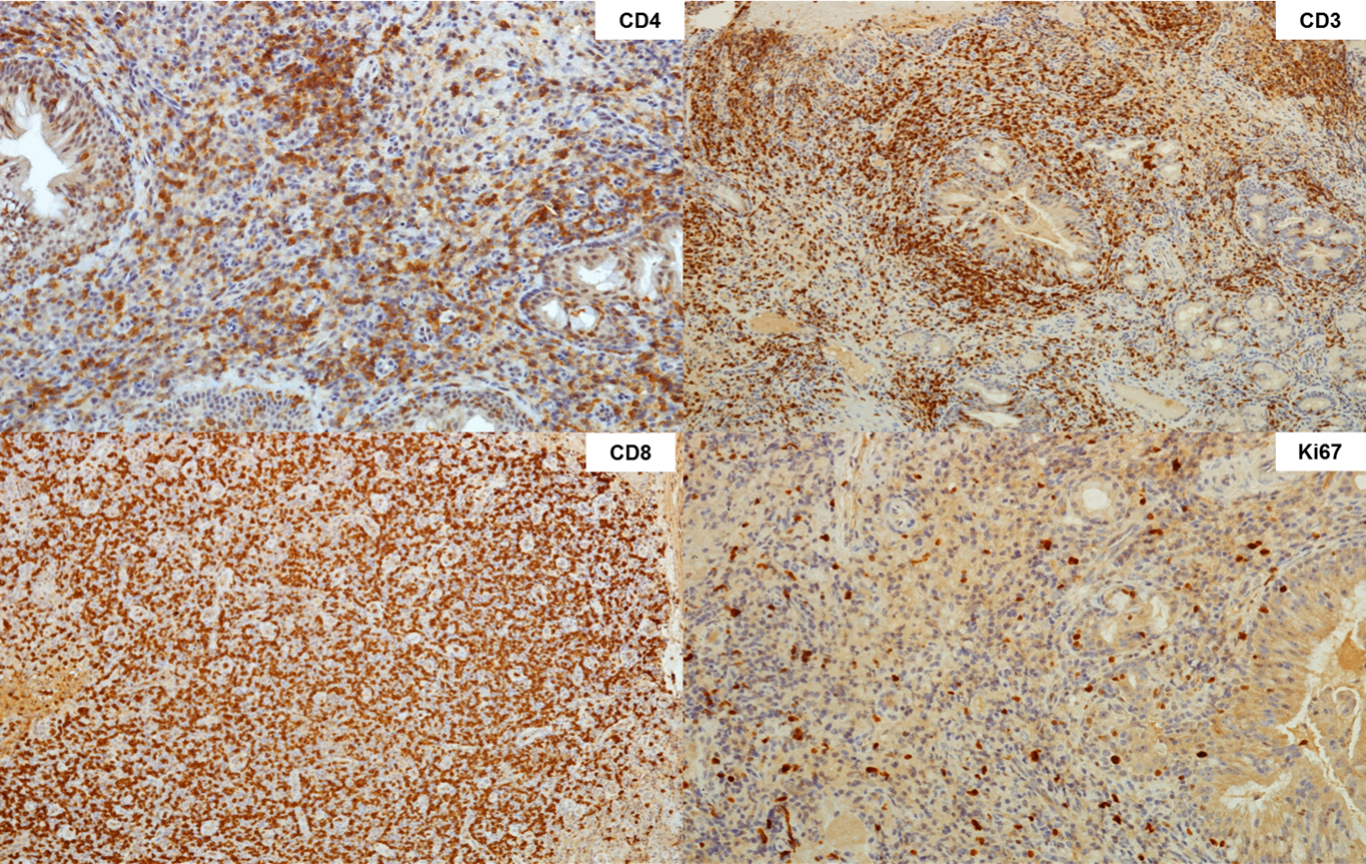
Immunohistochemistry showing monoclonal proliferation of T cells favoring the hypothesis of nasal lymphoma.

We herein report a case of a 37-year-old Brazilian man born in Minas Gerais, an endemic area of leishmaniasis in Brazil, who was receiving treatment with infliximab from 2005 to 2015 and since 2015 has been in use of adalimumab 40 mg every 2 weeks for ankylosing spondylitis. He presented to the otolaryngologist outpatient clinic of the University of São Paulo Medical School Hospital complaining of rhinorrhea and nasal obstruction for the last 3 months. Nasopharyngolaryngoscopy revealed a nasal granulomatous lesion with crust formation. The lesion was biopsied and initial hypotheses were leishmaniasis, lymphoma, syphilis, and Wegener granulomatosis. Antineutrophil cytoplasmic antibodies (ANCA) were negative. Serological assays for leishmaniasis were positive: ELISA with titers above 1:1280 and indirect immunofluorescence with a titer of 1:80. Hematoxylin and eosin staining showed a chronic lymphoplasmocytic infiltrate with ulcerated areas in the nasal mucosa (Fig 1). Amastigote forms were not visualized by optic microscopy. Polymerase chain reaction (PCR) was positive for *Leishmania* in the biopsy tissue [1]. Surprisingly, immunohistochemistry of the biopsy revealed CD3-positive, CD20-negative, CD56-negative, CD30-negative, Cyclin D1-negative, Ki-67-positive (high levels), CD4-positive (focal), CD8-positive (focal), CD7-positive, TIA-1-positive, and Granzyme B-positive cells, suggesting natural killer (NK)/T-cell lymphoma (Fig 2). The patient underwent a positron emission tomography (PET)/computed tomography (CT) scan, which was negative. CT scans of the sinus, chest, and abdomen were normal. In spite of the immunohistochemistry results, he was treated with a liposomal amphotericin B cumulative dose of 35 mg/kg [2], with complete remission of the lesions after 3 months.

Seven months after treatment, the patient returned with complaints of rhinorrhea, nose bleeding, and congestion. Coincidentally, he had been reinitiated on adalimumab 3 months earlier, prescribed by a rheumatologist. A new nasopharyngolaryngoscopy examination showed an ulcer with infiltrated borders in the left nasal septum. We decided on retreatment with 35 mg/kg of amphotericin B lipid complex. Four months after retreatment, the patient was asymptomatic and the ulcer had healed.

We decided to maintain secondary prophylaxis with 3 mg/kg of liposomal amphotericin B every 3 weeks for as long as the patient was on maintenance treatment with adalimumab.

## Discussion

The highlights of this article are the possibility of the reactivation of latent infections in patients receiving treatment with monoclonal antibodies for inflammatory and/or chronic autoimmune disease [3] and the need to consider these infections in the clinical and laboratorial differential diagnosis of tissue lesions in order to avoid misdiagnosis and wrong treatments.

Cutaneous and mucosal leishmaniasis usually induce a predominant type 1 immune response, characterized by high levels of interferon gamma (IFN-ɣ) and tumor necrosis factor alpha (TNF-α) [4]. Although associated with the control of infection, Th1 immune response can cause tissue damage if not modulated [5]. The parasite can persist in scars of old lesions for years, sustained by a persistent, well-modulated Th1 local immune response, even after treatment.

In mucosal leishmaniasis, TNF-α levels in the tissue are high before treatment and decrease after therapy [6]. The reactivation of latent infection is related to the deregulation of specific immune responses, decreasing inflammatory cytokines such as INF-ɣ and TNF-α associated with increasing Th2 cytokines interleukin 4 (IL4) and interleukin 10 (IL10) [7].

## Conclusion

Mucosal leishmaniasis should be part of the differential diagnosis of nasal lesions in patients on an immunosuppressive regimen, particularly those on anti-TNFα drugs [3, 8]. Diagnosis can be challenging, and the T-cell–mediated response induced by the parasite can render immunohistochemical analysis difficult, leading to misdiagnosis with nasal lymphoma.

Could the chronic inflammation triggered by mucosal leishmaniasis induce the development of lymphoma as can occur in the case of *Helicobacter pylori* infection and the development of mucosa-associated lymphatic tissue (MALT) lymphoma of the gastrointestinal tract [9, 10, 11]? We believe that the treatment of nasal leishmaniasis may be sufficient to improve local lymphoproliferation and to heal the lesion.

The negative CT and PET-CT scans, the positive PCR for *Leishmania*, and the healing of lesions after the 2 treatments reinforce and corroborate our hypothesis of mucosal leishmaniasis.

Once again, we emphasize the necessity of including latent infections in the investigation and differential diagnosis of lesions in patients on treatment with anti-TNFα drugs, mainly in those who live in or travel to endemic areas.

**Key learning points**

- The use of monoclonal antibodies against TNF can reactivate tegumentary leishmaniasis.
- The anatomopathological findings using the immunohistochemical method may sometimes lead to an error in the differential diagnosis between leishmaniasis and lymphoma.
- In cases of prolonged immunosuppression due to the use of monoclonal antibodies against TNF, secondary prophylaxis with liposomal amphotericin B may prevent relapses in mucosal leishmaniasis.

**Note:** The patient in this manuscript has given written informed consent (as outlined in the PLOS consent form) to publication of their case details.
